# Cutting-Edge Coronary Imaging Guiding CABG

**DOI:** 10.1177/15569845211008162

**Published:** 2021-04-20

**Authors:** Jasmin H. Shahinian, Aun Yeong Chong, David Glineur

**Affiliations:** 1 27339 Department of Cardiac Surgery, University of Ottawa Heart Institute, Canada; 2 Department of Cardiovascular Surgery, University Heart Center Freiburg, Bad Krozingen, Germany; 3 Department of Cardiology, University of Ottawa Heart Institute, Canada

## Introduction

Coronary artery disease (CAD) is one of the major causes of death in the worldwide population.^1^ Revascularization via percutaneous coronary intervention (PCI) or coronary artery bypass grafting (CABG) are current treatment modalities besides aggressive management of risk factors. Hence, accurate assessment and diagnosis of coronary artery disease is crucial in planning and implementing given treatment modality. Since the introduction of invasive coronary angiography (ICA) in 1958, it remains the most widely used modality to assess the anatomy and the extent of obstructive CAD.^2^ In fact, indication for CABG or PCI and pre-procedural planning are commonly based on visualization of CAD via ICA.^3,4^ While it allows assessment of coronary anatomy, degree of luminal obstruction, and blood flow, it is known to underestimate and/or overestimate lesion severity, especially for intermediate stenosis.^5^ The major reason for this inaccurate eyeballing estimation is the transformation of a 3-dimensional (3D) lesion into a 2-dimensional image. Moreover, there is a significant interindividual examiner variation in the degree of lesion estimation.^6^ Therefore, anatomical and morphological assessment of CAD is not only insufficient for understanding of the disease and coronary hemodynamics, but also for planning of complex interventions warranting additional functional and physiological assessment.

Recent advances in invasive and noninvasive cardiac imaging such as fractional flow reserve (FFR), instantaneous wave-free ratio (iFR), intravascular ultrasound (IVUS), optical coherence tomography (OCT), near-infrared spectroscopy, coronary computed tomography angiogram (CCTA), positron emission tomography (PET), and single-photon emission computerized tomography myocardial perfusion imaging (MPI) allow more accurate assessment of a given lesion directing correct indication and planning of a given procedure. While their utility has been studied to variable extents in the context of PCI, there is a paucity, and in some of the modalities, there is a total absence of data in CABG.

## 1. Quantitative Coronary Angiography

Quantitative coronary angiography (QCA) is based on contrast coronary angiography and was developed to objectify assessment of lumen diameter since the visual evaluation of coronary lesion during coronary angiography is prone to interobserver variability.^7^ In addition, it provides valuable information on the intermediate and long-term effects of coronary intervention. The technique of obtaining QCA has undergone several developments since its first validation study in 1977,^8^ and is currently performed using computer-generated automated edge detection systems. Parameters such as minimal luminal diameter (MLD), lesion length, reference vessel diameter, and diameter stenosis are obtained to assess luminal stenosis.^9^ While 3D QCA serves as a valuable tool to guide PCI, it has not been widely utilized in planning CABG strategy. Glineur et al. studied the correlation between the preoperative right coronary lesion MLD and the 6-month graft patency in a prospective randomized trial.^10^

## 2. Fractional Flow Reserve

FFR is a measurement of trans-stenotic pressure gradient allowing assessment of hemodynamic relevance of coronary artery stenoses. It is defined as the ratio between maximum achievable flow in a stenotic area to the theoretical maximum flow in a normal coronary artery.^11^ The measurement is carried out using a pressure wire, which is inserted across the coronary lesion. The ratio between mean coronary pressure distal to the stenosis to the mean aortic pressure during maximal hyperemia induced by adenosine infusion is measured.^12^

Since the introduction of FFR 25 years ago, its application has improved treatment strategy in patients with CAD becoming a standard reference when evaluating coronary lesions in the context of moderate disease and PCI. Utilization of FFR during PCI has found a broad platform with demonstrated improved clinical outcomes (FAME-2 Trial).^13^ A value of ≤0.8 indicates significant ischemia^14^ and is the current FFR cutoff for significant ischemia justifying the use of PCI. As with most functional measurements, FFR measurement has its pitfalls. Technical performance as well as the presence of another downstream stenosis, diffuse atherosclerosis, and/or microvascular disease can affect FFR measurement diverging from its actual value.^15^ Hence, its sole utilization to guide CABG remains questionable since several aspects including size and quality of diseased coronaries, present collateralizations, arrangement and geometry of grafts, and graft quality need to be considered. Moreover, the theoretical long-term protective effect of CABG could be lost due to the absence of grafting in case of negative FFR despite significant visual lesion estimation. Different types of studies have evaluated the use of FFR and CABG, graft patency or flow, and clinical outcome studies.

### FFR and Graft Patency or Flow

Botman et al.^16^ conducted a prospective study enrolling 164 patients who underwent CABG based on preoperatively measured FFR values. A follow-up angiography was conducted where 525 grafts were assessed. They demonstrated that bypassing lesions with FFR ≥0.75 resulted in higher rate of graft occlusion (21.4% in FFR >0.75 vs 8.9% in FFR <0.75; *P* < 0.001).

Honda et al.^17^ performed a retrospective study with a total of 72 patients evaluating the relationship between preoperatively measured FFR of left anterior descending (LAD) artery stenosis and the intraoperative flow pattern of the bypass graft. Patients were divided into 3 groups according to preoperative FFR values (Group S: FFR <0.70; Group M: 0.70 ≤ FFR <0.75; Group N: FFR ≥0.75). Graft patency was assessed intraoperatively using fluorescence imaging and postoperatively with coronary angiography or multislice CT within 1 year. In groups S, M, and N, the mean graft flow was 24.7, 19.2, and 16.0 ± 9.7 mL/minute (*P* = 0.009), and the pulsatility index was 2.35, 3.02, and 5.51 (*P* = 0.038), respectively. They concluded that as coronary stenosis severity increased, graft flow increased and pulsatility index decreased.

Glineur et al.^18^ conducted the Impact of Preoperative FFR on Arterial Bypass Graft Functionality (IMPAG) trial to evaluate the correlation between preoperatively measured FFR of the target vessel and anastomotic function 6 months after CABG. In total, 63 patients were enrolled, and 199 coronary lesions were evaluated. At 6 months, a systemic angiographic follow-up was performed to assess 199 arterial anastomoses. They found that arterial anastomoses performed to coronary arteries with an FFR value of <0.78 had a patency rate of 97%. They concluded that FFR was the predicting factor for arterial graft patency at 6 months and not angiographic stenosis severity.

In a substudy of the IMPAG trial,^19^ they also observed that a lower FFR cutoff of 0.71 has improved graft patency when bypassing the right coronary system taking into account the proportional relationship between the risk of competitive flow and the distance between graft inflow and distal coronary target.

### FFR and Clinical Outcome

Thuesen et al.^20^ evaluated the relationship between graft patency and clinical outcomes at 6 months in their Fractional Flow Reserve Versus Angiography Randomization for Graft Optimization (FARGO) trial. One hundred patients were enrolled in this trial and were randomly assigned to undergo either FFR-guided CABG where only targets with FFR <0.8 were bypassed or angiography-guided CABG where complete revascularization was performed. Seventy-five percent of grafts utilized in the trial were venous grafts accounting for all non-LAD grafts. Angiographic follow-up was performed at 6 months. There was no difference in graft patency observed between the 2 groups. Moreover, there were no differences in the rates of myocardial infarction, stroke, revascularization, and death at 1 year. The following limitations of the FARGO trial warrant caution in data interpretation. First, the study was stopped prematurely at 58% of the expected enrollment. Second, 12% of patients did not undergo the planned treatment. Third, 25% of patients failed to undergo follow-up angiography. Lastly, the observed rates of graft occlusion after 6 months with FFR >0.8 and FFR <0.8 were 10% and 8%, respectively, compared to the predicted rate of 20% and 5%, respectively, providing the basis for power calculation.

The Graft Patency After FFR-guided Versus Angiograph-guided CABG (GRAFFITI)^21^ trial, a prospective randomized trial, including 172 patients examined the impact of FFR on graft patency and clinical outcomes. Patients were randomized to undergo angiography-guided or FFR-guided CABG with an FFR cutoff set at 0.8. The ratio of arterial versus venous grafts was 1:1. Angiography follow-up was performed at 1 year. There was no difference in graft patency or in major adverse cardiac events (MACE) observed at 1 year. Several limitations of this trial merit caution in interpreting results. First, 33% and 37% of patients in the angiography-guided and FFR-guided groups failed to undergo angiographic follow-up at 1 year. Second, the trial was stopped at only 83.5% of the cohort size due to slow enrollment. Third, surgeon reluctance to change CABG strategy according to FFR might have diminished the significance of FFR guidance. In fact, in the FFR-guided group, 29% of the deferred vessels had FFR <0.8 and 11% of bypassed vessels had FFR >0.8. Lastly, the trial was possibly underpowered for clinical and angiographic outcomes.

Toth et al.^22^ and Fournier et al.^23^ conducted a retrospective study aiming to investigate the impact of FFR-guided CABG versus angiography-guided CABG on long-term clinical outcomes. The study enrolled 667 patients, with 429 patients undergoing angiography-guided CABG and 198 patients undergoing FFR-guided CABG with at least 1 stenosis grafted according to FFR. Stenoses with an FFR value of >0.80 were deferred for revascularization. Graft patency was assessed during angiographic follow-up at 6 years. Patency of 327 arterial (64%) and 185 venous grafts (36%) was assessed. At 6 years, 15% graft occlusion was observed with occlusion rates of venous graft being higher than the rate of occluded arterial grafts with 24% and 10%, respectively (*P* < 0.001). Considering overall graft occlusion in both groups, the proportion of graft occlusion was lower in the FFR-guided group with 9% and 17%, respectively (*P* = 0.024). Moreover, FFR guidance was the only predicting factor of graft patency in arterial grafts. As for the long-term clinical outcome, the rate of MACE (log rank: 0.209; *P* = 0.238), death (log rank: 0.111; *P* = 0.137), and myocardial infarction (log rank: 0.082; *P* = 0.092) was lower in the FFR-guided group at 6 years as compared with the angiography-guided group.

### FFR and CABG Summary

With the results and the significant bias in the 2 prospective randomized trials (FARGO and GRAFFITI), it remains challenging to implement the standard application of FFR to guide CABG. Its utilization in CABG remains low in spite of Class 1a recommendation for intermediate stenosis when noninvasive testing failed to demonstrate ischemia.^24^ Based on the current evidence and the absence of data from larger trials powered to detect differences in clinical outcomes, the use of FFR as the only guide to dictate whether a patient should become a candidate for surgical revascularization strategy versus PCI and/or which vessels should or should not be grafted should be discouraged. However, FFR seems to be an important tool to help decide which type of grafts to use (arterial for lesions with an FFR <0.78, venous for those with higher values).

## 3. Instantaneous Wave-Free Ratio

iFR is another physiological index to evaluate the significance of coronary artery stenosis and is computed by measuring the resting pressure gradient across a coronary stenosis during diastole without requiring hyperemia.^25^ It is especially useful in assessing serial or diffuse coronary disease to characterize the functional relevance of a given stenosis.^26^ A co-registration system of iFR pullback during angiography has been developed to pinpoint the predominant lesion in tandem stenoses. This can further facilitate revascularization strategies during PCI.^27^ While iFR is known to be more sensitive in the detection of critical stenoses than FFR, FFR accounts not only for the myocardial size supplied by the given diseased coronary artery but also for the quality of the bypassed vessels and the presence of distal disease. Evidence on the role of FFR and/or iFR in guiding CABG strategies remains limited with only a few studies addressing FFR correlation to graft patency, functionality, and clinical outcome^28^ warranting further studies on the topic.

## 4. FFR Cardiac CT

FFR derived from cardiac CT (FFR-CT) is a recent noninvasive modality, which is obtained from CTA images using computational fluid dynamics.^29^ It enables accurate assessment of CAD incorporating anatomic and physiologic data, providing FFR measurement at any individual lesion and along the entire course of the coronary tree without the need for provocative maneuvers as in the invasive measurement of FFR. Hence, interpretation of FFR-CT warrants caution, since it is rather a dynamic value subjected to the location of the measurement, present atherosclerosis, and collaterals.^30^ Similar to iFR with angiography co-registration plotting the values along the vessel, there are currently no data available to guide the position of the distal anastomosis and whether it actually matters for CABG strategy planning. Min et al. compared invasively derived FFR with FFR-CT at a given lesion within the coronary vessel demonstrating a high correlation^31^ with the same cutoff value of ≤0.8 indicative of myocardial ischemia. Moreover, the addition of FFR-CT is a promising tool in the noninvasive identification of significant coronary lesions providing valuable insight into vessel disease anatomy and physiology. Its introduction into clinical practice has the potential to improve the efficiency of therapeutic strategies. However, while current studies report high accuracy and specificity for FFR-CT including intermediate coronary stenosis, there are not sufficient data for recommendations in clinical practice.

## 5. FFR_angio_

FFR_angio_ is a recent development based on coronary angiography images where FFR is derived using proprietary software eliminating the need for pressure wire and adenosine. FFR_angio_ software algorithm allows 3D reconstruction of the entire coronary tree, coronary vessel section, and color-code the FFR reading for the given lesion to aid assessment of culprit lesion. Voiding the need for pressure-wire enables a quick assessment of the physiologic significance of the stenosis. A multicenter, prospective, international trial (FFR_angio_ Accuracy versus Standard FFR [FAST-FFR])^32^ was performed evaluating the accuracy of FFR_angio_ compared to pressure-wire derived FFR. Ten centers enrolled a total of 301 patients. Mean FFR was 0.81, and 43% of vessels had an FFR of ≤0.80. The per-vessel sensitivity and specificity were 94% (95% confidence interval [CI], 88%-97%) and 91% (95% CI, 86%-95%), respectively. FFR_angio_ values correlated well with FFR measurements (*r* = 0.80, *P* < 0.001) with diagnostic accuracy of FFR_angio_ at 92% overall. Authors concluded that FFR_angio_ had high sensitivity and specificity allowing accurate assessment of FFR. Clinical trials on utilizing FFR_angio_ in guiding revascularization strategies are required for further evaluation.

## 6. Noninvasive Coronary Tests

Another noninvasive detection of obstructive CAD offers PET with qualitative and quantitative assessment of myocardial perfusion. Evaluation of coronary flow reserve (CFR) defined as the ratio between myocardial blood flow at peak hyperemia and at rest allows quantification of functional severity. Lower CFR values <1.5 have been reported to be associated with increased risk of cardiac death.^33^ Moreover, Taqueti et al. showed that when CFR was <1.5, patients who underwent CABG had lower MACE compared to PCI or conservative therapy.^34^

Although several studies report an ischemic cutoff threshold between 1.4 and 2.5, there is no current consensus on a clear value. While PET MPI is a valuable resource providing a physiological assessment of myocardial dysfunction and ischemic severity, additional studies will be necessary to establish its role in guiding revascularization decisions.

## 7. IVUS and OCT

Further invasive testing modalities such as IVUS and OCT have been brought forward in guiding CABG. Both modalities have a greater spatial resolution as compared to angiography providing additional information on the morphology of the coronary artery lesion. IVUS imaging allows real-time evaluation of vessel size, lumen area, plaque composition, and volume due to its deeper tissue penetration.^35^ Whereas OCT, with its light-based source, provides precise identification of intraluminal structures requiring blood-free field. While it is superior to IVUS in acute intraprocedural assessment, its limited penetration is prone to underestimating plaque burden and miscalculating vessel size.^36^ Available studies on IVUS and OCT guidance during PCI suggest better intraprocedural outcomes, such as stent evaluation, repeat revascularization, residual thrombosis, and/or dissection as well as long-term patient outcome as compared to angiography-guided PCI. There is limited evidence on the utilization of intraluminal imaging techniques such as IVUS and OCT in guiding CABG. While current guidelines state a Class 2a recommendation to assess the severity of unprotected left main lesions, there is a potential application for intravascular imaging for evaluation of intermediate stenosis.^37^

## Conclusions

More advanced coronary imaging is an adjunct tool to guide CABG strategy and evaluate its long-term outcome. While invasive coronary angiography remains the gold standard in the assessment of stenosis severity, novel invasive and noninvasive techniques provide additional functional and physiological information to optimize treatment strategy. Additional studies utilizing these modalities would be necessary to institute their role in coronary revascularization guidelines.
